# Mechanisms of Isoprene Decoupling in Poplar: Precursor Dynamics and VOC Fluxes Under Acute Thermal Exposure and Elevated CO_2_

**DOI:** 10.3390/plants15081196

**Published:** 2026-04-14

**Authors:** Miguel Portillo-Estrada

**Affiliations:** Research Group PLECO (Plants and Ecosystems), Department of Biology, University of Antwerp, 2610 Antwerp, Belgium; miguel.portilloestrada@uantwerpen.be

**Keywords:** biogenic volatile organic compounds (BVOCs), dimethylallyl diphosphate (DMADP), elevated CO_2_, ethanol, heat stress, isoprene, lipoxygenase (LOX) products, methanol, photosynthesis, reactive oxygen species (ROS)

## Abstract

Rising temperatures and atmospheric CO_2_ exert complex, interacting effects on plant carbon metabolism and volatile organic compound (VOC) emissions. This study investigated the physiological mechanisms underlying acute thermal tolerance in *Populus nigra* by integrating leaf gas exchange with high-resolution proton-transfer-reaction time-of-flight mass spectrometry (PTR-TOF-MS). We employed a factorial design (25–40 °C; 400 and 800 ppm CO_2_) to examine how metabolic regulation and pulse-induced signalling interact across thermal gradients. Our results identify a critical metabolic tipping point around 40 °C, representing a transition toward a survival-orientated state. Isoprene emission decoupled from net photosynthesis at this threshold; while carbon assimilation collapsed, isoprene was maintained at near-maximal rates to prioritize thylakoid thermal protection. Under moderate temperatures (25–35 °C), emission capacity scaled linearly with the chloroplastic DMADP pool, but this relationship broke down at 40 °C. Notably, elevated CO_2_ sustained the magnitude of stress-related “bursts” at the thermal limit, suggesting that increased carbon availability provides the metabolic stamina required to fuel emergency defence and fermentative pathways. These findings demonstrate that acute thermal exposure triggers a metabolic reconfiguration, shifting resources from growth-oriented processes toward survival-based stabilization mechanisms.

## 1. Introduction

Plants face continual exposure to fluctuating environmental conditions that disrupt carbon assimilation and metabolic homeostasis. Among abiotic stressors, high light, elevated temperature, and rising atmospheric CO_2_ exert profound and interacting effects on leaf metabolism, often leading to photoinhibition, oxidative damage, and reduced growth [1,2]. Understanding how plants tolerate these stressors is therefore essential for predicting ecosystem responses to climate change, particularly in fast-growing genera such as *Populus*. Members of this genus dominate large areas of temperate and boreal forests and represent a major global source of volatile organic compound (VOC) emissions [3,4].

Ongoing climate warming is intensifying heat stress in these ecosystems and triggering a range of physiological adjustments, including altered chloroplastic carbon allocation, membrane peroxidation, and oxidative bursts. These processes are frequently reflected in changes in volatile organic compound (VOC) emissions, which may function both as protective metabolites and as indicators of cellular damage [5,6]. In *Populus*, constitutive isoprene emissions—synthesized in chloroplasts via the methylerythritol phosphate (MEP) pathway from dimethylallyl diphosphate (DMADP) linked to photosynthetic electron transport—enhance thermotolerance by stabilizing thylakoid membranes and quenching reactive oxygen species (ROS) [1,6,7]. Emission rates respond strongly to light and temperature and can become partially decoupled from net photosynthesis under stress, with emissions persisting or even increasing despite declining photosynthetic rates during heatwaves or drought [1,2,8]. This behaviour suggests that isoprene biosynthesis may increasingly reflect metabolic adaptation to acute heat—shifting from a supply-driven process linked to carbon assimilation toward a demand-driven protective mechanism—although the regulation of this transition under interacting global change drivers remains moderately understood [9,10].

Isoprene emission is primarily governed by biochemical rather than diffusive controls and depends largely on the availability of its precursor DMADP and the catalytic activity of isoprene synthase (IspS) [1,11,12]. Under favourable conditions, emission rates typically scale with IspS activity when precursor pools are abundant. Increasing temperature, however, can alter the balance between precursor supply and enzymatic capacity within the MEP pathway, potentially shifting regulatory control between substrate availability and enzyme kinetics [13,14]. Although elevated CO_2_ concentrations generally suppress isoprene emission at moderate temperatures, likely through changes in carbon allocation and MEP pathway regulation, its influence under extreme thermal treatments—when internal carbon pools may be rapidly mobilized—remains less well understood [3,13,15]. Clarifying how precursor pools, enzyme activity, and photosynthetic performance interact across temperature and CO_2_ gradients is therefore essential for predicting isoprene emissions under future climatic conditions [9,10].

A useful in vivo probe of substrate regulation is post-illumination isoprene emission. Following darkening, transient isoprene release reflects the residual DMADP pool remaining once photosynthesis ceases [11,12]. This approach, validated in *Populus* and other isoprene emitters, provides a practical proxy for estimating chloroplastic precursor availability under changing environmental conditions [2]. Such measurements allow researchers to distinguish whether temperature-driven changes in isoprene emission arise primarily from alterations in enzymatic capacity or from shifts in precursor supply.

Beyond isoprene, several stress-related VOCs—including lipoxygenase (LOX) pathway products (green leaf volatiles, GLVs), methanol, acetone, reactive carbonyls, short-chain organic acids, and ethanol—serve as indicators of oxidative stress, membrane disruption, and metabolic reconfiguration [5,16,17]. In drought-stressed *Populus*, these compounds often increase alongside isoprene emissions, providing complementary biomarkers of physiological thresholds and broader metabolic adaptation [18,19,20]. LOX-derived GLVs reflect lipid peroxidation and membrane damage [21,22]; methanol originates from pectin demethylation during cell wall remodelling as well as damage-related processes [22,23]; and ethanol indicates fermentative metabolism under hypoxia or severe energetic imbalance [24,25]. These emissions are often amplified under combined heat and drought stress and may signal the transition from regulated physiological adjustment toward cellular dysregulation. In such cases, crossing a critical hygrothermal threshold triggers a broader reconfiguration of the leaf volatile profile [26]. Furthermore, the magnitude of these stress-induced bursts may be contingent upon internal carbon availability, where elevated CO_2_ potentially supports sustained secondary metabolic flux even as primary assimilation fails. However, these diverse emissions are rarely integrated into functional biosynthetic groups to evaluate metabolic thresholds in *Populus*, limiting our ability to interpret VOC signals as mechanistic biomarkers of cellular dysregulation [26].

In this study, we develop an integrated framework linking photosynthetic metabolism and VOC emissions in *Populus nigra* L. Building on previous observations of temperature-driven decoupling between isoprene emission and carbon assimilation, we examined how metabolic regulation and stress signalling interact across temperature gradients and contrasting CO_2_ conditions. We tested four hypotheses:

**Hypothesis** **1 (H1).** *Isoprene emission progressively decouples from net photosynthesis at supra-optimal temperatures, signalling a metabolic shift in which carbon is increasingly prioritized for membrane stabilization and thermal protection rather than primary assimilation.*

**Hypothesis** **2 (H2).** *The capacity for isoprene emission is governed primarily by the chloroplastic pool size of its precursor, dimethylallyl diphosphate (DMADP), such that emission rates scale with substrate availability across temperature and CO_2_ gradients.*

**Hypothesis** **3 (H3).** *While elevated CO_2_ is known to suppress isoprene at moderate temperatures via biochemical downregulation of the MEP pathway, this effect is overridden during acute thermal exposure as internal carbon mobilization bolsters precursor supply.*

**Hypothesis** **4 (H4).** *Stress-induced VOCs serve as diagnostic biomarkers for cellular damage thresholds; however, elevated CO_2_ does not merely “buffer” these bursts but shapes their trajectory, providing the metabolic resilience and substrate required to sustain secondary pathways during the thermal crisis.*

By integrating isoprene dynamics with broader stress-VOC signatures, this work aims to refine mechanistic biomarkers of acute thermal adaptation in *Populus* and improve the interpretation of plant VOC signals for predicting ecosystem responses to concurrent increases in temperature and atmospheric CO_2_.

## 2. Materials and Methods

### 2.1. Plant Material and Gas Exchange Measurements

*Populus nigra* L. (Brandaris genotype) clone cuttings were planted in 20 L pots, in a soil composed of 90% sand and 10% peat by volume, with constant drip watering. One month prior to the experiment, the potted saplings were transferred to a growth room with a 16 h photoperiod, diurnal temperature regime (22 °C/16 °C night), 400 ppm CO_2_ and relative humidity of approximately 40–60%. During this period, plants were maintained under LED grow light bars (Mezzo 85W, TotalGrow, Alvin, TX, USA), providing a photosynthetic photon flux density (PPFD) of ca. 400 µmol photons m^−2^ s^−1^ at the canopy level. The plants were watered manually to pot capacity thrice a week, or as needed, to ensure constant hydration. Nutritional requirements were managed through the initial incorporation of a balanced, slow-release fertilizer (15:9:12 N:P:K + micronutrients) at a dosage of 4 g L^−1^, providing a steady supply of essential macro- and micronutrients. Further details regarding the plant material are provided in [20].

Measurements were conducted on fully expanded, mature leaves from sun-exposed branches of the study trees. Only healthy leaves without visible damage, pathogen infection, or signs of senescence were selected. To minimize developmental variability, leaves were chosen from the mid-canopy and from branches exposed to similar light conditions (ca. 250 µmol photons m^−2^ s^−1^). Each leaf was used for a single experimental treatment combination.

Leaf photosynthetic activity, stomatal conductance, and volatile organic compound (VOC) emissions were measured simultaneously using a LI-6400XT portable photosynthesis system (LI-COR Biosciences, Lincoln, NE, USA) coupled online to a proton-transfer-reaction time-of-flight mass spectrometer (PTR-TOF-MS; model 8000, Ionicon Analytik GmbH, Innsbruck, Austria). A sample air bypass flow of 74 µmol s^−1^ exiting the leaf cuvette was diverted to the PTR-TOF-MS. Following the setup and processing protocols described in [20], the drift tube was operated at 600 V, 2.5 mbar, and 60 °C, resulting in a reduced electric field of ≈120 Td (1 Townsend = 10^−17^ V cm^2^).

Raw data were recorded at 1 s time resolution. More information about peak fitting, software, VOC identification and reaction rate constants can be found in [20]. Detailed information regarding instrument calibration and the discrimination of adjacent peaks can be found in [22,27].

To ensure saturating conditions for net photosynthesis measurements, preliminary light response curves were derived to determine the optimal photosynthetically active radiation (PAR). Consequently, PAR was maintained between 1000 and 1600 μmol photons m^−2^ s^−1^ for all temperature and CO_2_ combinations.

Measurements were conducted using the LI-6400XT system, equipped with a 6 cm^2^ chamber and an integrated LED source (LI-6400-02B). This system was used to regulate leaf temperature and CO_2_ concentration while maintaining a constant flow rate of 500 μmol s^−1^ and ca. 60% relative humidity.

### 2.2. Experimental Treatments

The experiment employed a 4 × 2 factorial design to evaluate metabolic shifts in *Populus* leaves from optimal growth conditions to acute thermal treatments. Measurements were conducted at four steady-state leaf temperatures (25, 30, 35, and 40 °C) under two atmospheric CO_2_ concentrations: ambient (400 µmol mol^−1^) and elevated (800 µmol mol^−1^), yielding eight treatment combinations. Leaves were allowed to acclimate to each temperature/CO_2_ combination for 20–30 min until gas exchange parameters and isoprene emissions reached a stable steady state before recording data.

This temperature range was selected to transition from the thermal optimum for *Populus nigra* photosynthesis (25–30 °C) to the onset of metabolic dysregulation and potential structural damage at 40 °C [20,28]. The manipulation of CO_2_ concentration was specifically employed to evaluate two competing physiological mechanisms: the CO_2_-induced biochemical inhibition of the MEP pathway (H3) and the capacity for elevated CO_2_ to provide metabolic buffering against thermal exposure (H4).

A total of 48 leaves from six independent plants were used (six biological replicates per treatment). To minimize genetic variability and ensure reproducibility, all plants were genetically identical clones derived from the same parent stock. Each leaf was assigned to a single CO_2_ and temperature combination to avoid carryover effects. All measurements were conducted during midday (10:00–15:00 h) to ensure stable diurnal metabolic activity.

### 2.3. In Vivo Isoprene Precursor Dynamics and Photosynthetic Capacity

To determine the maximum photosynthetic capacity (*A*_net_), light response curves were derived at each temperature and CO_2_ treatment level, with *A*_net_ defined as the net CO_2_ assimilation rate under saturating light conditions.

The chloroplastic pool size of dimethylallyldiphosphate (DMADP) and the apparent isoprene synthase (IspS) activity were determined in vivo using the light-to-dark transition method as described by [29,30]. This approach is based on the metabolic decay of isoprene emission following the sudden cessation of the methylerythritol phosphate (MEP) pathway flux by sharply applying darkness to the leaves. Briefly, upon reaching steady-state isoprene emission at the light level that yielded maximum photosynthetic capacity (*A*_net_) at a given temperature and CO_2_, the actinic light was abruptly shut off. The resulting post-illumination isoprene decay was monitored at 1 s intervals until a stable dark baseline was reached.

The total DMADP pool size was estimated by integrating the area under the isoprene decay curve, representing the exhaustion of the pre-existing substrate reservoir accumulated during the light period. The apparent IspS activity was derived from the initial decay rate, reflecting the enzymatic capacity to convert the available substrate pool into emission under the prevailing leaf conditions.

All emission data were corrected for background VOC concentrations by performing regular empty-chamber blank measurements under identical environmental conditions.

### 2.4. Classification and Identification of VOC Groups

To evaluate the physiological transition of poplar leaves from steady-state metabolism (25 °C) to acute thermal exposure (40 °C), six groups of VOCs were categorized based on their biosynthetic origins and roles as stress biomarkers:

- Isoprene (C_5_H_9_^+^, *m*/*z* 69.070): Used as the primary indicator of chloroplastic carbon allocation and photosynthetic activity. Produced via the MEP pathway, its emission reflects the availability of photosynthetic precursors (G3P and pyruvate) and contributes to thermal protection by stabilizing thylakoid membranes under heat stress [26,31].
- Lipoxygenase (LOX) pathway products: Green leaf volatiles (GLVs) were monitored as indicators of membrane damage. Identified via C_6_H_11_O^+^ (*m*/*z* 99.080; hexenals), C_6_H_13_O^+^ (*m*/*z* 101.096; hexanal and hexenols), C_8_H_15_O_2_^+^ (*m*/*z* 143.107; hexenyl acetate), and C_5_H_11_O^+^ (*m*/*z* 87.080; pentanal and 1-penten-3-ol). GLVs are markers for lipid peroxidation or thermal disruption of cellular membranes [32,33].
- Reactive oxidative carbonyls: Markers of oxidative stress and enhanced photorespiration. This group includes C_2_H_2_O^+^ (*m*/*z* 45.034; acetaldehyde), C_3_H_7_O^+^ (*m*/*z* 59.049; propanal/acetone), C_4_H_9_O^+^ (*m*/*z* 73.065; butanal/methyl ethyl ketone), and isoprene oxidation products C_4_H_7_O^+^ (*m*/*z* 71.049; MVK/MACR), which serve as internal markers for the interaction between isoprene and reactive oxygen species within the leaf. Additional markers included C_3_H_7_O_2_^+^ (*m*/*z* 75.045; hydroxyacetone), C_2_H_3_O_2_^+^ (*m*/*z* 59.014; glyoxal), and C_3_H_5_O_2_^+^ (*m*/*z* 73.028; methylglyoxal). These compounds arise when elevated temperatures reduce *Rubisco* specificity, favouring oxygenation and promoting ROS-mediated degradation of carbon intermediates [34,35,36].
- Methanol (CH_5_O^+^, *m*/*z* 33.034): Indicator of cell wall modification. Released during pectin demethylation by pectin methylesterase (PME), a process that accelerates during cell wall expansion or thermal degradation of pectic polysaccharides [37,38].
- Short-chain organic acids: CH_3_O_2_^+^ (*m*/*z* 47.013; formic acid) and C_2_H_5_O_2_^+^ (*m*/*z* 61.028; acetic acid) were monitored as indicators of metabolic overflow. These C_1_–C_2_ acids are released when glycolytic flux exceeds the capacity of the TCA cycle under stress or through hydrolysis of acetyl groups from hemicellulose at high temperature [39].
- Ethanol (C_2_H_7_O^+^, *m*/*z* 47.049): Marker of internal hypoxia. Under high temperatures, mitochondrial oxygen demand may exceed the capacity of stomatal and internal diffusion, forcing a shift toward ethanolic fermentation to sustain ATP production under oxygen limitation [25].

### 2.5. Statistical Analysis

Overall metabolic trends were modelled according to the regressions in Table S1 (Supplementary Materials), with independent two-tailed Student’s *t*-tests used to resolve statistical differences between CO_2_ treatments at each discrete temperature (Table S2; Supplementary Materials).

## 3. Results and Discussion

### 3.1. Photosynthetic Control and Isoprene Carbon Investment

**Figure 1:**
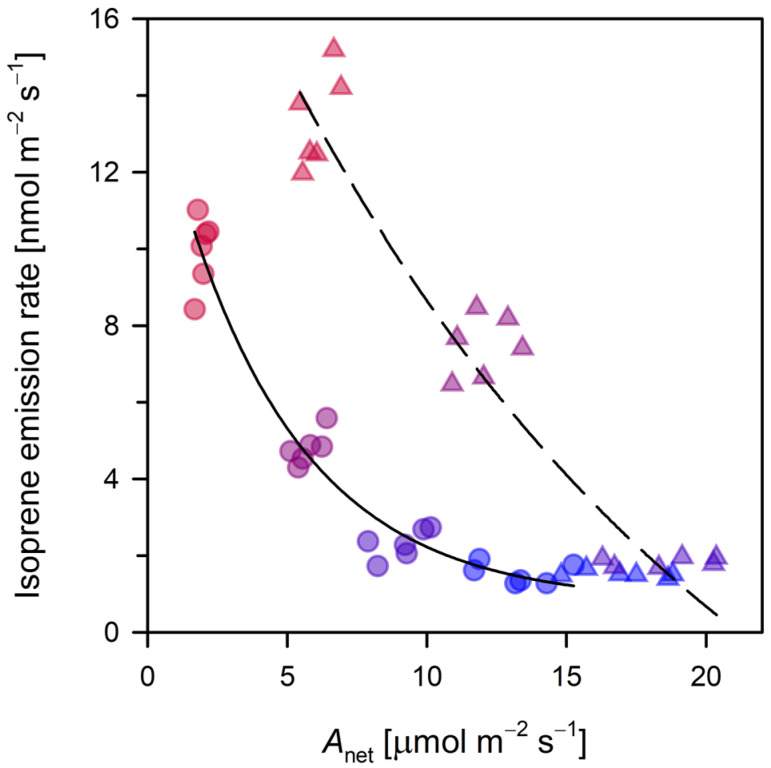
Photosynthetic assimilation (*A*_net_; µmol CO_2_ m^−2^ s^−1^) and isoprene emission (nmol m^−2^ s^−1^) under ambient (400 ppm, circles, solid) and elevated (800 ppm, triangles, dashed) CO_2_. Data points are color-coded from blue to red to depict increasing leaf temperature (25–40 °C). Fitting parameters are provided in Table S1, and *p*-values from Student’s *t*-tests comparing the two CO_2_ levels are listed in Table S2 (Supplementary Materials).

At the control temperature of 25 °C, gas exchange parameters indicate a state of constitutive metabolism. While elevated CO_2_ (800 ppm) enhanced the maximum assimilation rate (*A*_net_) by approximately 30% compared with ambient levels, isoprene emission remained stable at 1.5 nmol m^−2^ s^−1^ (Figure 1), suggesting that CO_2_-mediated inhibition is negligible at thermal optima in this clone. In this optimal thermal range, poplar leaves maintain steady carbon allocation between growth and protective volatile synthesis, with minimal stress-induced shifts [11].

Divergence emerges as temperatures rise to 35–40 °C. Contrary to the common observation of CO_2_ suppression, elevated CO_2_ significantly stimulated isoprene emissions, which reached 13.3 nmol m^−2^ s^−1^ at 40 °C (a 34% increase over ambient treatments; Figure 1). This “release” from inhibition confirms that high temperatures functionally override the instantaneous CO_2_ sensitivity typically observed at cooler temperatures [40]. As previously observed in *Populus* under interactive stressors, the suppressive effect of elevated CO_2_ is secondary to the thermal stimulation of the MEP pathway [13]. Mechanistically, this likely reflects a shift in which increased carbon availability from elevated CO_2_ bolsters the chloroplastic precursor supply (pyruvate and G3P), sustaining a higher flux even as photosynthetic efficiency begins to decline. This pattern provides a nuanced confirmation of (H3), indicating that CO_2_ regulation is not a fixed constraint but is dynamically modulated by thermal exposure.

**Figure 2:**
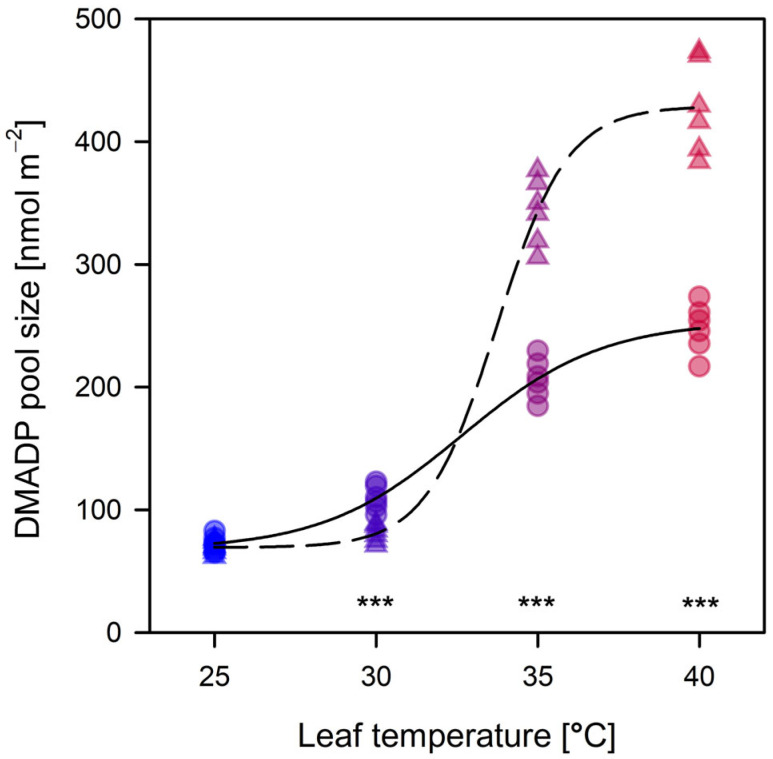
Temperature response of dimethylallyl diphosphate (DMADP) pool sizes (nmol m^−2^). Leaf-level DMADP pools were measured in *Populus nigra* across a temperature gradient under ambient (400 ppm, circles, solid) and elevated (800 ppm, triangles, dashed) CO_2_ concentrations. Data points are color-coded from blue to red to depict increasing leaf temperature (25–40 °C). Fitting parameters (sigmoidal regressions) are provided in Table S1, and *p*-values from Student’s *t*-tests comparing the two CO_2_ levels are listed in Table S2 (Supplementary Materials). Asterisks (***) denote *p* < 0.001.

At the 40 °C thermal extreme, a profound shift occurred as maximum *A*_net_ collapsed to near-minimal levels (<2 µmol CO_2_ m^−2^ s^−1^ under ambient CO_2_, Figure 1) due to thermal inhibition of *Rubisco* and electron transport [8,41]. In contrast, isoprene emissions peaked dramatically, achieving complete decoupling from current photosynthesis [9,42]. The expected CO_2_ inhibition faded at this threshold, replaced instead by a stimulatory effect, indicating a fundamental shift in metabolic regulation. This uncoupling supports the thermal protection hypothesis (H1), as isoprene’s role in thylakoid membrane stabilization and ROS quenching becomes paramount, overriding the suppressive mechanisms observed at milder temperatures [43,44]. This maintenance of high flux occurred alongside the saturation of the DMADP pool (Figure 2), indicating that, at 40 °C, the system transitions from substrate-limited to enzyme-limited kinetics, where IspS activity becomes the primary bottleneck. Plants likely mobilize internal carbon stores, such as carbon from photorespiratory intermediates from redirected metabolic pathways (e.g., fermentation and organic acid accumulation), to sustain isoprene production for short-term survival amid photosynthetic shutdown [9,45,46]. This metabolic transition reflects a prioritized allocation of internal carbon toward protective VOCs and fermentative pathways, effectively pausing growth-related assimilation in favour of acute physiological survival. Although sustained exposure to 40 °C may eventually exceed the leaf’s homeostatic capacity, the maintenance of dynamic, non-zero metabolic fluxes during our experimental window indicates a regulated adaptive state aimed at preserving leaf integrity rather than the onset of immediate necrotic collapse.

### 3.2. Thermal Threshold of the DMADP Precursor Pool

The DMADP pool exhibited a consistent temperature-dependent expansion, increasing progressively from 25 °C to a maximum observed magnitude at 40 °C (Figure 2). Contrary to expectations of metabolic exhaustion, the precursor pool did not decline at the thermal extreme; instead, it reached its highest concentrations under acute heat stress. At 35 °C, leaves under 800 ppm CO_2_ maintained significantly higher DMADP levels than those at 400 ppm (≈343 vs. ≈203 nmol m^−2^), a trend that intensified at 40 °C, at which the 800 ppm treatment reached a peak of ≈430 nmol m^−2^ (Figure 2).

This sustained accumulation indicates that higher carbon availability at elevated CO_2_ supports an increasingly larger precursor reservoir within the methylerythritol phosphate (MEP) pathway, even as primary carbon fixation declines [47]. This accumulation is likely fuelled by a transition in carbon sourcing; at extreme temperatures, the contribution of non-photosynthetic carbon precursors—encompassing mobilized starch reserves, cytosolic sugars, and vacuolar pools of organic acids (e.g., malate, citrate) and amino acids (e.g., alanine)—increases significantly to maintain isoprene biosynthesis despite the collapse of current assimilation [15].

This trend provides strong validation for (H3), as CO_2_ concentration clearly dictates the magnitude of the carbon supply to the MEP pathway. Statistical analyses confirmed this CO_2_-driven divergence across all temperature treatments (*p* < 0.001), emphasizing the regulatory role of atmospheric carbon on precursor pools. Notably, the surge in DMADP at 40 °C occurred concurrently with peak isoprene emissions (Figure 2), suggesting that, at this threshold, the rate of precursor replenishment from internal carbon stores exceeds the rate of enzymatic consumption by isoprene synthase [48].

The resulting accumulation suggests that, under acute heat stress, the leaf transitions into a “high-pressure” metabolic state. Rather than a metabolic drawdown, this state of precursor saturation ensures that isoprene production is not substrate-limited during the thermal crisis, supporting the thermal protection hypothesis (H1) [30]. The leaf effectively prioritizes flooding the MEP pathway to preserve thylakoid integrity, even as photosynthetic metabolism (*A*_net_) becomes increasingly constrained.

### 3.3. Decoupling Between DMADP Availability, Isoprene Synthase Activity and Isoprene Emission at High Temperature

**Figure 3:**
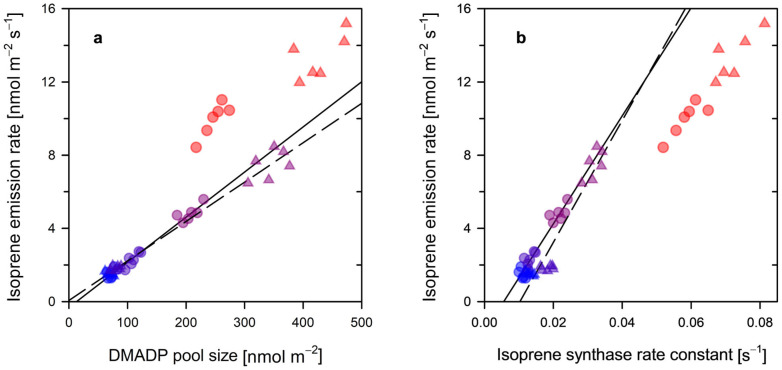
Biochemical regulation of isoprene emission (nmol m^−2^ s^−1^) under ambient (400 ppm, circles, solid) and elevated (800 ppm, triangles, dashed) CO_2_: Panels show: (**a**) relationship with DMADP pool size (nmol m^−2^); and (**b**) relationship with the isoprene synthase (IspS) rate constant (s^−1^). Values at 40 °C (red symbols) are excluded from the linear regressions to emphasize the metabolic decoupling from the standard metabolic flux control. Data points are color-coded from blue to red to depict increasing leaf temperature (25–40 °C). Fitting parameters are provided in Table S1, and *p*-values from Student’s *t*-tests comparing the two CO_2_ levels are listed in Table S2 (Supplementary Materials).

Across the 25–35 °C temperature range, isoprene emission showed a robust positive relationship with both its immediate chloroplastic precursor and enzymatic driver. At both 400 and 800 ppm CO_2_, emission rates increased linearly with both the dimethylallyldiphosphate (DMADP) pool size (Figure 3a) and the apparent isoprene synthase (IspS) rate constant (Figure 3b) [12,30]. This linear coupling reinforces the metabolic flux control hypothesis (H2), demonstrating how isoprene emission is governed by a coordinated increase in substrate availability and enzymatic capacity under non-inhibitory conditions [30,49]. The similarity in regulatory patterns between CO_2_ treatments in this range suggests that the fundamental biochemical control of the methylerythritol phosphate (MEP) pathway remains conserved until thermal thresholds are reached.

However, a clear decoupling emerged at 40 °C (Figure 3a,b), characterized by a transition to a high-velocity kinetic state in which the linear scaling of (H2) breaks down. At this thermal extreme, the apparent IspS rate constant deviated from the moderate trajectories of lower temperatures, surging to values between 0.06 and 0.08 s^−1^. This suggests that, as the leaf approaches its thermal limit, the enzyme enters a high-velocity mode, likely reflecting the intrinsic high-temperature optimum (45–50 °C) of the isoprene synthase enzyme. This kinetic surge at 40 °C suggests that the leaf has transitioned out of its hormetic window—in which thermal effects might still be compensatory—into a state of metabolic distress. At this thermal extreme, the breakdown of the linear scaling observed at lower temperatures indicates that the regulatory mechanisms are no longer maintaining homeostatic balance but are instead operating in an emergency mode to prevent immediate thermal collapse.

Interestingly, while isoprene emissions peaked significantly (13.3 nmol m^−2^ s^−1^ at 800 ppm), the DMADP pool did not deplete; rather, it reached its maximum observed magnitude (430–470 nmol m^−2^). This indicates that, at 40 °C, the decoupling is not caused by precursor limitation but by an overwhelming push from internal carbon reserves that floods the MEP pathway even as photosynthesis (*A*_net_) crashes. The breakdown of the previous linear relationships provides biochemical evidence that acute thermal exposure overrides typical metabolic constraints [50]. This “super-exponential” behaviour of the IspS rate constant ensures that every available molecule of DMADP is rapidly converted to isoprene, prioritizing thylakoid stabilization (H1) and ROS quenching during the photosynthetic shutdown [9,42,49]. The loss of CO_2_ sensitivity at this threshold further confirms that the leaf has transitioned from a steady-state supply regime to an emergency, exhaustive utilization of metabolic buffers to ensure short-term survival.

### 3.4. Heat-Induced Shifts in Stress-Related VOC Pathways

#### 3.4.1. Threshold Dynamics of Secondary Metabolism

**Figure 4:**
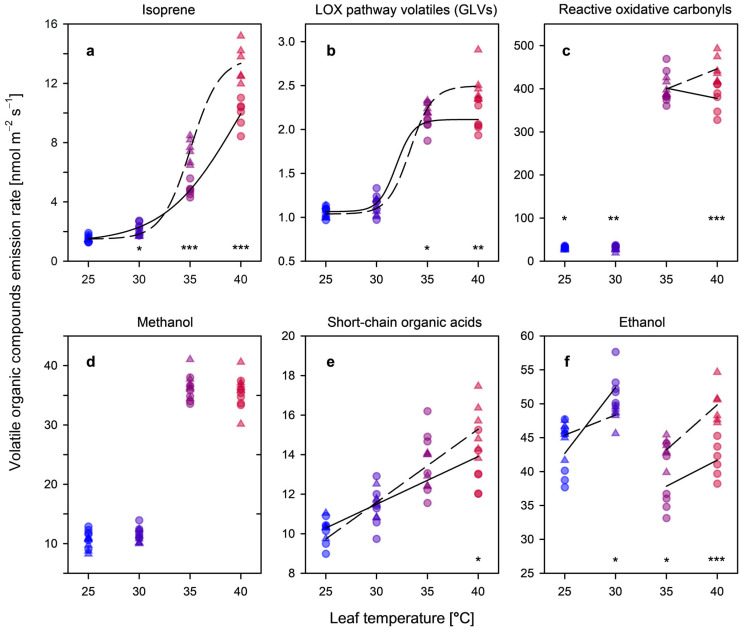
Temperature-dependent VOC emission profiles at ambient (400 ppm, circles, solid) and elevated (800 ppm, triangles, dashed) CO_2_. Panels show: (**a**) isoprene; (**b**) LOX pathway products (C6 aldehydes: hexanal, *(Z)*-3-hexenal, *(E)*-2-hexenal; C6 alcohols: *(Z)*-3-hexenol, *(E)*-2-hexenol; C6 esters: *(Z)*-3-hexenyl acetate); (**c**) reactive oxidative carbonyls (formaldehyde, acetaldehyde, acetone, and methylglyoxal); (**d**) methanol (marker for cell-wall pectin demethylation); (**e**) short-chain organic acids (formic and acetic acid); and (**f**) ethanol (anaerobic fermentation marker). Fitting parameters and significance levels are provided in Table S1 (Supplementary Materials). Significant differences between CO_2_ treatments at specific temperatures are denoted by asterisks: * *p* < 0.05, ** *p* < 0.01, *** *p* < 0.001 (Student’s *t*-test). Detailed *p*-values are provided in Table S2.

The transition from moderate temperatures (25–35 °C) to acute thermal exposure (40 °C) induced a pronounced reconfiguration of the leaf volatile profile, marking a critical metabolic threshold between steady-state homeostatic regulation and an emergency, stress-driven reconfiguration [5,26,51]. This transition represents the boundary of the hormetic window, where the metabolic focus shifts from growth-oriented maintenance to acute thermal preservation. This progression is initially characterized by the sigmoidal response of isoprene (Figure 4a), which reaches a plateau at 40 °C, signalling the exhaustion of constitutive protective capacity. While most other pulse-responsive VOCs remained near baseline levels within the moderate temperature range (25–30 °C), exposure to 40 °C triggered a coordinated, multi-fold increase across several volatile pathways, indicating widespread structural perturbation of leaf metabolism [5,51,52].

Importantly, the interaction between temperature and CO_2_ revealed a distinct shift in metabolic behaviour. Rather than uniformly suppressing stress responses, elevated CO_2_ intensified the magnitude of several thermal-induced emissions at 40 °C, particularly reactive carbonyls and short-chain organic acids (Figure 4c,e). This pattern suggests that leaves grown under elevated CO_2_ may retain greater metabolic turnover at the thermal limit, allowing continued processing of internal carbon pools and sustaining both protective pathways, such as isoprene biosynthesis, and stress-associated metabolic responses (H4) [53,54].

#### 3.4.2. Lipoxygenase Pathway Products (GLVs)

Green leaf volatiles (GLVs), related derivatives generated via the lipoxygenase (LOX) pathway, responded strongly to extreme temperature (Figure 4b). At 40 °C under 400 ppm CO_2_, LOX-derived emissions increased markedly, reflecting rapid lipid peroxidation of membrane polyunsaturated fatty acids [52]. Interestingly, the 800 ppm treatment displayed a sustained emission magnitude (≈2.38 nmol m^−2^ s^−1^) compared with ambient conditions. This increase may indicate that enhanced carbon availability under elevated CO_2_ supports continued metabolic flux through the LOX pathway even under severe thermal exposure. Such behaviour suggests sustained metabolic activity rather than the partial metabolic inhibition often observed under extreme stress in carbon-limited tissues [5].

#### 3.4.3. Reactive Oxidative Carbonyls

Reactive carbonyl compounds, including acetaldehyde, acetone, and methylglyoxal, remained close to detection limits between 25 and 35 °C but increased sharply at 40 °C (Figure 4c). These compounds are commonly associated with reactive oxygen species (ROS) formation and oxidative degradation of metabolic intermediates under stress conditions [34,55]. In contrast to the expectation that elevated CO_2_ might suppress oxidative metabolism through reduced photorespiration, the highest carbonyl flux was observed under 800 ppm CO_2_ (≈451 nmol m^−2^ s^−1^). This pattern suggests that leaves under elevated CO_2_ may maintain higher metabolic turnover and higher oxidative processing capacity under acute heat stress, whereas the lower emissions at 400 ppm CO_2_ could reflect substrate depletion or reduced metabolic activity under severe physiological limitation [55].

#### 3.4.4. Methanol: Cell Wall Remodelling and Damage

Methanol emissions, primarily produced through pectin demethylation catalysed by pectin methylesterases (PMEs), increased markedly between 35 and 40 °C (Figure 4d). A relatively high constitutive baseline of methanol emission (≈11 nmol m^−2^ s^−1^) was observed across treatments, consistent with the active cell wall metabolism characteristic of *Populus*. At the highest temperature, emissions converged between CO_2_ treatments (≈36 nmol m^−2^ s^−1^), suggesting that, once the thermal threshold is exceeded, structural processes associated with cell wall modification or degradation proceed largely independently of carbon supply [26,56].

#### 3.4.5. Short-Chain Organic Acids

Emissions of short-chain organic acids, particularly formic and acetic acid, increased substantially at 40 °C, reflecting a major disruption of primary carbon metabolism (Figure 4e) [39]. These emissions were highest under 800 ppm CO_2_, reaching peak values of ≈15.4 nmol m^−2^ s^−1^. Such increases likely reflect a metabolic overflow metabolism under severe stress conditions, in which glycolytic flux and carbohydrate breakdown exceed the processing capacity of mitochondrial respiration [39]. Under elevated CO_2_, greater internal carbon availability appears to promote the accumulation and emission of these acidic metabolites during photosynthetic inhibition [5,20].

#### 3.4.6. Ethanol: Indicator of Fermentative Metabolism

Ethanol emissions served as a clear indicator of the transition toward fermentative metabolism (Figure 4f) [25]. The response followed a biphasic pattern: at 30 °C, elevated CO_2_ suppressed the moderate ethanol increase observed under ambient conditions, suggesting improved metabolic balance at intermediate temperatures. However, at 40 °C, ethanol emissions increased sharply under elevated CO_2_ (≈49.9 nmol m^−2^ s^−1^), likely reflecting the activation of anaerobic pathways to sustain ATP production under thermal constraint [24,25].

This rise suggests an activation of anaerobic fermentation pathways to sustain minimal ATP production during photosynthetic inhibition. Consistent with the mobilization of internal carbon stores observed in other *Populus* species [15], the stronger response under elevated CO_2_ indicates that greater availability of non-photosynthetic carbohydrate reserves allows for a sustained fermentative flux during acute heat stress. This substrate-enabled “emergency metabolism” may provide a critical energetic bridge [9], supporting cellular maintenance when primary aerobic pathways are thermally constrained.

### 3.5. Synthesis of the Systemic Metabolic Response to Extreme Heat

**Figure 5:**
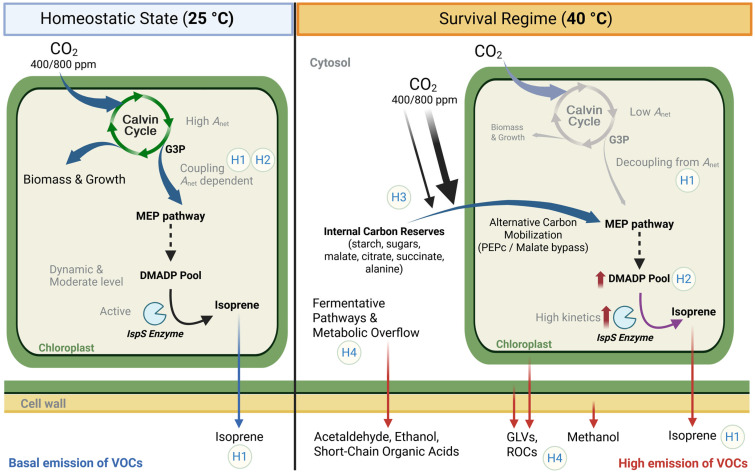
Conceptual model of isoprene decoupling and stress-induced VOC signatures in *Populus nigra* at the thermal threshold. The diagram illustrates the systemic metabolic reconfiguration occurring at the 40 °C critical thermal tipping point, separating the (**left**) homeostatic state (25 °C) from the (**right**) survival regime (40 °C). Homeostatic State (25 °C): Under optimal conditions, metabolism is growth-oriented. Net photosynthetic assimilation (*A*_net_) via the Calvin cycle is high and dynamic, providing a steady supply of G3P (glyceraldehyde 3-phosphate) and pyruvate to the chloroplastic MEP (methylerythritol 4-phosphate) pathway. Isoprene emission (blue arrow towards outside the cell) is tightly coupled (H1) to recent photosynthate, with IspS (isoprene synthase) operating under constitutive kinetics and a dynamic, non-saturated DMADP (dimethylallyl diphosphate) precursor pool. Survival Regime (40 °C): At the thermal limit, a transition to emergency metabolism occurs. (H1) Decoupling: Primary assimilation collapses (*A*_net_); however, isoprene emission is maintained through the mobilization of internal carbon reserves (starch, sugars, malate, citrate, succinate, alanine) from the cytosol and vacuoles. (H2) Precursor saturation: High-temperature optimum kinetics of the IspS enzyme, combined with a saturated DMADP pool, sustain near-maximal emission rates (red arrow) despite the cessation of photosynthesis. (H3) Metabolic stamina: Elevated CO_2_ (800 ppm) acts as a metabolic safeguard under high temperature, fuelling an alternative carbon mobilization route via PEPc (phosphoenolpyruvate carboxylase). This process facilitates a Malate bypass, in which cytosolic carboxylation provides the necessary substrates and energetic stamina to the MEP pathway to preserve thylakoid integrity. (H4) Stress-VOC signatures: The breakdown of cellular regulation is evidenced by the emission (red arrows) of diverse VOCs (volatile organic compounds). These include biomarkers of integrity loss: GLVs (green leaf volatiles, originating from membrane lipid peroxidation), ROCs (reactive oxidative carbonyls, indicating severe oxidative stress and ROS—reactive oxygen species—formation), and methanol (released from pectin demethylation in cell walls). Emergency fermentative pathways: The “metabolic overflow” of alternative carbon leads to the emission of acetaldehyde, ethanol, and short-chain organic acids (e.g., acetic and formic acid), serving as indicators of a regulated but non-sustainable survival strategy.

The systemic metabolic shifts occurring at the 40 °C threshold are integrated into a conceptual model (Figure 5), illustrating the transition from primary carbon fixation to an emergency “survival regime” sustained by internal carbon mobilization (H1) and precursor pool saturation (H2). This framework highlights how elevated CO_2_ facilitates a metabolic bypass (H3) while concurrent stress-VOC emissions—such as methanol, GLVs, and reactive carbonyls—signal the onset of cellular dysregulation (H4).

## 4. Conclusions

This study identifies a critical metabolic threshold at 40 °C in *Populus nigra*, marking a systemic transition from homeostatic regulation to an emergency, survival-driven regime. Our findings demonstrate that isoprene decoupling from photosynthesis is not a sign of metabolic exhaustion or the onset of an unregulated apoptotic pathway but a regulated adaptive shift supported by saturated chloroplastic precursor pools and sustained high-velocity IspS kinetics (H1, H2). This mechanism prioritizes thylakoid protection through the rapid mobilization of internal carbon reserves, effectively maintaining leaf viability during acute heat stress. Notably, the reversal of the typical CO_2_-inhibition effect at 40 °C reveals that elevated CO_2_ acts as a metabolic fuel rather than a constraint at this thermal extreme (H3), while the coordinated bursts of diverse VOC classes serve as physiological biomarkers for this regulated metabolic reconfiguration (H4).

Together, these results identify a physiological tipping point at which metabolism diverts from growth-supporting pathways toward an acute survival-oriented state focused on maintaining cellular integrity. While this reconfiguration facilitates immediate thermal stabilization and preserves transient viability, we acknowledge that sustained incubation at 40 °C would likely exceed the leaf’s repair capacity, eventually leading to a loss of viability. Incorporating these non-steady-state precursor dynamics and metabolic thresholds into emission models is essential for accurately predicting forest–atmosphere interactions in a warming, CO_2_-enriched future.

## Data Availability

The data presented in this study are openly available at Zenodo https://doi.org/10.5281/zenodo.19550809.

## Supplementary Materials

The following supporting information can be downloaded at https://www.mdpi.com/article/10.3390/plants15081196/s1: Table S1: Regression parameters for metabolic models; Table S2: Matrix of *p*-values for CO_2_ treatment effects.
