# Supplementary material for: Mechanisms of Isoprene Decoupling in Poplar: Precursor Dynamics and VOC Fluxes Under Acute Thermal Exposure and Elevated CO2
Source: Plants (Basel). 2026 Apr 14;15(8):1196. doi: 10.3390/plants15081196 (PMC13120222; doi:10.3390/plants15081196)
Supplement: Supplementary file 1 [file plants-15-01196-s001.zip › plants-4230987-supplementary.pdf]

Mechanisms of Isoprene Decoupling in Poplar: Precursor Dynamics and VOC Fluxes under Acute Thermal Exposure and Elevated CO_2

Miguel Portillo-Estrada

Supplementary Materials

Table S1. Summary of regression equations describing the relationships presented in Figures 1 through 4. Data are categorized by CO_2 treatment (400 and 800 ppm).

Figure	Data	Regression type	Title 2	Title 3
Figure 1 (400 ppm)	All	Exponential decay	y = 0.77+14.19*exp(–0.228x)	R^2 = 0.950; P < 0.001
Figure 1 (800 ppm)	All	Exponential decay	y = –9.92+ 32.62*exp(–0.056x)	R^2 = 0.911; P < 0.001
Figure 2 (400 ppm)	All	Sigmoidal	y = 67.333+186.60/(1+exp(–(x–32.66)/2.160))	R^2 = 0.984; P < 0.001
Figure 2 (800 ppm)	All	Sigmoidal	y = 69.270+359.69/(1+exp(–(x–33.73)/1.090))	R^2 = 0.950; P < 0.001
Figure 3a (400 ppm)	25-35 °C	Linear	y = –0.317 + 0.0247x	r^2 = 0.976; P < 0.001
Figure 3a (800 ppm)	25-35 °C	Linear	y = –0.059 + 0.0216x	r^2 = 0.979; P < 0.001
Figure 3b (400 ppm)	25-35 °C	Linear	y = –1.646 + 294.8x	r^2 = 0.934; P < 0.001
Figure 3b (800 ppm)	25-35 °C	Linear	y = –3.424 + 333.0x	r^2 = 0.920; P < 0.001
Figure 4a (400 ppm)	All	Sigmoidal	y = 1.254+16.72/(1+exp(–(x–39.71)/3.592))	R^2 = 0.976; P < 0.001
Figure 4a (800 ppm)	All	Sigmoidal	y = 1.495+12.24/(1+exp(–(x–35.06)/1.428))	R^2 = 0.980; P < 0.001
Figure 4b (400 ppm)	All	Sigmoidal	y = 1.065+1.049/(1+exp(–(x–31.93)/0.858))	R^2 = 0.948; P < 0.001
Figure 4b (800 ppm)	All	Sigmoidal	y = 1.037+1.458/(1+exp(–(x–33.31)/1.090))	R^2 = 0.971; P < 0.001
Figure 4c (400 ppm)	35-40 °C	Linear	y = 568 – 4.766x	r^2 = 0.101; P = 0.316
Figure 4c (800 ppm)	35-40 °C	Linear	y = 71.5 + 9.369x	r^2 = 0.507; P = 0.009
Figure 4d (400 ppm)		NA		
Figure 4d (800 ppm)		NA		
Figure 4e (400 ppm)	All	Linear	y = 4.254 + 0.241x	r^2 = 0.508; P < 0.001
Figure 4e (800 ppm)	All	Linear	y = 0.470 + 0.371x	r^2 = 0.811; P < 0.001
Figure 4f (400 ppm)	25-30 °C	Linear	y = –6.009 + 1.948x	r^2 = 0.679; P = 0.010
Figure 4f (800 ppm)	25-30 °C	Linear	y = 30.56 + 0.593x	r^2 = 0.461; P = 0.015
Figure 4f (400 ppm)	35-40 °C	Linear	y = 10.88 + 0.770x	r^2 = 0.255; P = 0.094
Figure 4f (800 ppm)	35-40 °C	Linear	y = –3.320 + 1.329x	r^2 = 0.700; P < 0.001

Table S2. Values represent p-values derived from independent two-tailed t-tests comparing ambient (400 ppm) and elevated (800 ppm) CO_2 treatments at each temperature level (n = 6 per treatment). A_{net}: maximum net photosynthetic assimilation; DMADP: chloroplastic dimethylallyl diphosphate; IspS: apparent isoprene synthase rate constant; LOX: lipoxygenase pathway products. Bold values indicate statistical significance at α = 0.05.

				VOC emissions					
Leaf Temperature	A_{net}	DMADP pool	IspS rate constant	Isoprene	LOX products	Reactive oxidative carbonyls	Methanol	Short-chain organic acids	Ethanol
25	**0.0013**	0.383	**0.0071**	0.850	0.440	**0.047**	0.069	0.729	0.197
30	**<0.001**	**<0.001**	**<0.001**	**0.018**	0.352	**0.004**	0.061	0.719	**0.011**
35	**<0.001**	**<0.001**	**<0.001**	**<0.001**	**0.032**	0.920	0.188	0.579	**0.020**
40	**<0.001**	**<0.001**	**<0.001**	**<0.001**	**0.006**	**0.004**	0.501	**0.018**	**<0.001**
